# Aberrant expression of KPNA2 is associated with a poor prognosis and contributes to OCT4 nuclear transportation in bladder cancer

**DOI:** 10.18632/oncotarget.11889

**Published:** 2016-09-07

**Authors:** Jingcheng Zhou, Daoquan Dong, Ran Cheng, Yan Wang, Shuqi Jiang, Yuhong Zhu, Longlong Fan, Xiangming Mao, Yaoting Gui, Zesong Li, Xianxin Li, Bentao Shi

**Affiliations:** ^1^ Department of Urology, Peking University Shenzhen Hospital, Shenzhen, Guangdong 518036, China; ^2^ Guangdong and Shenzhen Key Laboratory of Male Reproductive Medicine and Genetics, Institute of Urology, Peking University Shenzhen Hospital, Shenzhen PKU-HKUST Medical Center, Shenzhen, Guangdong 518036, China; ^3^ Shenzhen Sun Yat-sen Cardiovascular Hospital, Shenzhen, Guangdong 518112, China; ^4^ Shenzhen Key Laboratory of Genitourinary Tumor, Shenzhen Second People's Hospital, First Affiliated Hospital of Shenzhen University, Shenzhen, Guangdong 518035, China

**Keywords:** KPNA2, OCT4, bladder cancer, prognostic, nucleo-cytoplasmic transport

## Abstract

Recent studies show that Karyopherin alpha 2 (KPNA2) is up-regulated in quite a number of cancers and associated with poor prognosis. Here, we found that expression levels of KPNA2 and OCT4 are up-regulated in bladder cancer tissues and significantly associated with primary tumor stage and bladder cancer patients' poorer prognosis. Our data also showed decreased cell proliferation and migration rates of bladder cancer cell lines when the expression of KPNA2 and OCT4 was silenced. Meanwhile, cell apoptosis rate was increased. Furthermore, Co-IP and immunofluorescence assay showed the KPNA2 interacts with OCT4 and inhibits OCT4 nuclear transportation when KPNA2 was silenced. Thus, we confirmed that up-regulated KPNA2 and OCT4 expression is a common feature of bladder cancer that is correlated with increased aggressive tumor behavior. Also, we propose that KPNA2 regulates the process of OCT4 nuclear transportation in bladder cancer.

## INTRODUCTION

Bladder cancer occupied the first position of incidence and mortality among genitourinary tumors in china [1]. The incidence of bladder cancer is increasing in most areas of the world for which statistics are available for recent decades [2, 3]. The high mortality rate of bladder cancer is in part due to lack of early detection of biological marker [4]. One of the important reasons is lack of full understanding of the mechanism to treat bladder cancer. Recent studies have shown that the development of bladder cancer is regulated by multi-gene interaction, which contains a multitude of complex process [5, 6].

KPNA2 is a protein which has a joint nuclear localization signal region, in mediating the nuclear translocation of a variety of important proteins in cells. Expression of KPNA2 has an impact on the relevant function by regulating protein subcellular localization, and takes part in signal transduction from the extracellular stimulus to the nucleus in the process [7]. In our previous study, we found that for upper tract urothelial carcinoma (UTUC) patients, who have received radical nephroureterectomyh (RNU) operation, high expression of KPNA2 indicates poorer prognosis and could be used as an independent marker for bladder recurrence, overall survival(OS) and disease-free survival(DFS). Moreover, KPNA2 may be a potential target for the treatment of UTUC [8]. UTUC is rare and accounts for only 5–10% of urothelial carcinoma while bladder cancer takes up about 90–95% [9]. Bladder cancer may share some risk factors with UTUC. In primary diagnosed UTUC patients, the possibility of concurrent bladder cancer is up to 33% [10]. However, the mechanism of KPNA2 in the development of bladder cancer is still unknown and needed to be further studied.

Given the role of nuclear transport factor, KPNA2 was reported having a feature of bounding with its cargo proteins and forms a ternary complex [11]. Hence, KPNA2-bind cargo protein may synergize with KPNA2 in the occurrence and development of tumor. It is reported that the interaction between Oct4 and karyopherin-alpha 2 may be contributed to the nuclear localization of Oct4 [12]. Furthermore, KPNA2 overexpression interacted with OCT4 to promote cell proliferation in non-small cell lung cancer (NSCLC) [13]. However, the physiological roles of KPNA2 and the interaction between KPNA2 and OCT4 in bladder cancer cells has not been investigated to date.

Here, we report that KPNA2 and OCT4 protein levels are frequently increased in bladder cancer clinical tissues and bladder cell line and associated with bladder cancer clinic-pathological parameters. Moreover, the effect of KPNA2 on cell proliferation, apoptosis and migration of bladder cancer cell lines has been analyzed. Furthermore, we showed that OCT4 interacted with KPNA2 and demonstrate that KPNA2 is involved in the nuclear transport process of OCT4.

## RESULTS

### Up-regulated expression of KPNA2 and OCT4 in bladder cancer clinical tissues and cell lines is correlated with clinical pathological features

**Figure 1:**
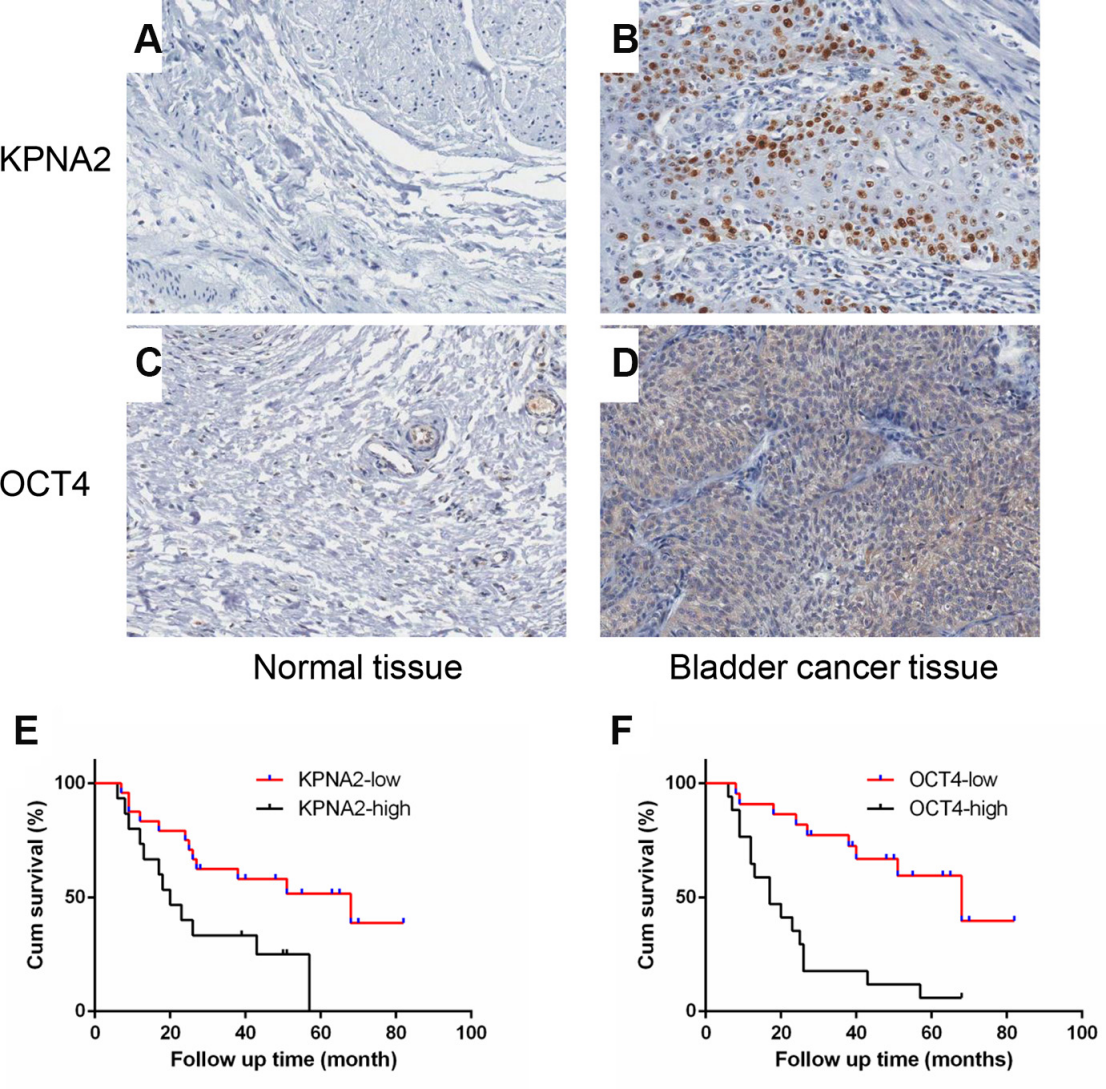
KPNA2 and OCT4 are up-regulated in bladder cancer tissues and its association with prognosis (**A**–**D**) Representative IHC staining for KPNA2 and OCT4 expression in bladder cancer tissues and normal bladder tissues. (**E**, **F**) The cumulative survival rate was higher in the low protein expression group compared with high KPNA2 or OCT4 expression group.

The protein expression of KPNA2 and OCT4 were detected by using the immunohistochemistry(IHC) in a total of 195 bladder cancer clinical tissues. In particular, only 39 clinical samples were paired with adjacent normal tissues and accompanied with follow up time data. Among the 195 bladder cancer samples, interestingly, the IHC results showed that the KPNA2 protein was mainly detected in the cell nucleus, with few positive immunostaining areas in cytoplasm, while the OCT4 protein were primarily expressed in cytoplasmic of cancer cells (Figure 1A–1D). The Spearman's correlation coefficient showed that expression levels of KPNA2 was positively correlated with levels of OCT4 (*r* = 0.257, *P* < 0.001). The correlation between KPNA2, OCT4 protein expression level and clinical pathological factors was indicated in Table 1. As summarized in Table 1, the patients were assigned into four groups according to the expression levels of KPNA2 and OCT4. Up-regulated KPNA2 and OCT4 expression were positively correlated with primary tumor stage and pathological types. No significant correlations were observed between the expression level and gender, age.

**Table 1:** High expression level of KPNA2 and OCT4 are correlated with clinical pathological features in bladder cancer

	No. of Cases	KPNA2 expression		χ^2^	*P*	OCT4 expression		χ^2^	*P*
		low	high			low	high		
All cases	195	132	63			79	116		
**Gender**									
Male	153	100	53	1.307	0.253	61	92	0.030	0.863
Female	42	32	10			18	24		
**Age**									
≤ 60	90	60	30	0.017	0.897	40	50	0.790	0.374
> 60	105	72	33			39	66		
**Primary tumor stage**									
T1	74	58	16	13.049	0.001*	44	30	21.388	< 0.001*
T2	92	62	30			31	61		
T3	29	12	17			4	25		
**Pathological type**									
Urothelial carcinoma, grade I	58	57	1	41.351	< 0.001*	30	28	8.721	0.033*
Urothelial carcinoma, grade II	66	41	25			22	44		
Urothelial carcinoma, grade III	32	12	20			8	24		
Other types	39	22	17			19	20		

* *P* < 0.05 was considered to be stoically significant.

We examined the correlation between the expression levels of KPNA2 or OCT4 with the bladder cancer prognosis using 39 samples mentioned above (Figure 1E–1F). As shown in the overall survival curve, the patients in high-KPNA2 expression group had a significantly poorer prognosis compared with low-KPNA2 group (*P* = 0.0195), as well as the high OCT4 group (*P* = 0.0002). The median survival time for the patients in the KPNA2 low expression group was 68 months when compared with 20 months for those in the KPNA2 high expression group. Taken these results together, we found that the high expression of KPNA2 and OCT4 were independent prognostic predictors for bladder cancer patients.

### Expression of KPNA2 and OCT4 was knocked down by specific siRNAs

**Figure 2:**
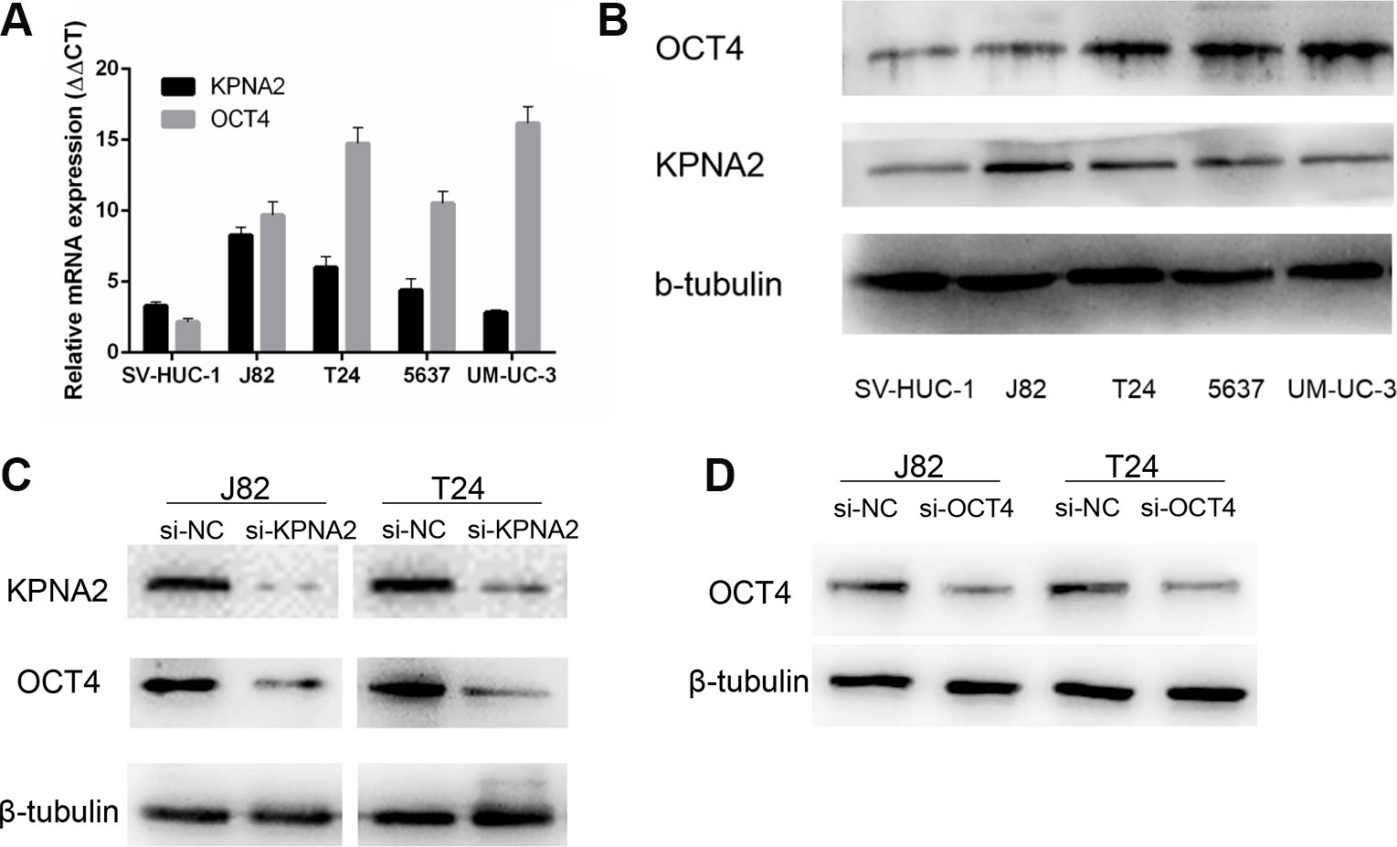
The mRNA and protein expression level of KPNA2 and OCT4 in bladder cancer cell lines and their changes after transfection with respective siRNAs (**A**) Relative *KPNA2* and *OCT4* mRNA levels were up-regulated in bladder cancer cell lines compared with human uroepithelial cell line. (**B**) KPNA2 and OCT4 presented higher protein expression in four bladder cancer cell lines. (**C**, **D**) Western Blot results indicated KPNA2 and OCT4 specific siRNAs significantly down-regulate the protein expression levels.

To confirm the knockdown efficiency of the specific siRNAs, we first detected the endogenic expression level of KPNA2 and OCT4 in bladder cancer cell lines J82, T24, 5637, UM-UC-3 and human uroepithelial cell line SV-HUC-1 through RT-qPCR and Western-blot analysis. The KPNA2 and OCT4 expression level in each cell line were indicated in Figure 2A–2B. As is shown in Figure 2A and 2B, KPNA2 and OCT4 were both up-regulated in J82 and T24 cell lines when compared with SV-HUC-1. Based on these results, we choose J82 and T24 to investigate the potential effects of KPNA2 and OCT4 on bladder cells *in vitro*. J82 and T24 were cultured and treated with KPNA2-siRNA and negative control siRNA. At 48h after transfection, the protein expression level of KPNA2 were significantly down-regulated by the specific siRNA relatively (Figure 2C). Moreover, we found that the expression level of OCT4 were downregulated simultaneously when KPNA2 was silenced (Figure 2C).

### Silencing KPNA2 or OCT4 can induce cell apoptosis and inhibit cell proliferation

**Figure 3:**
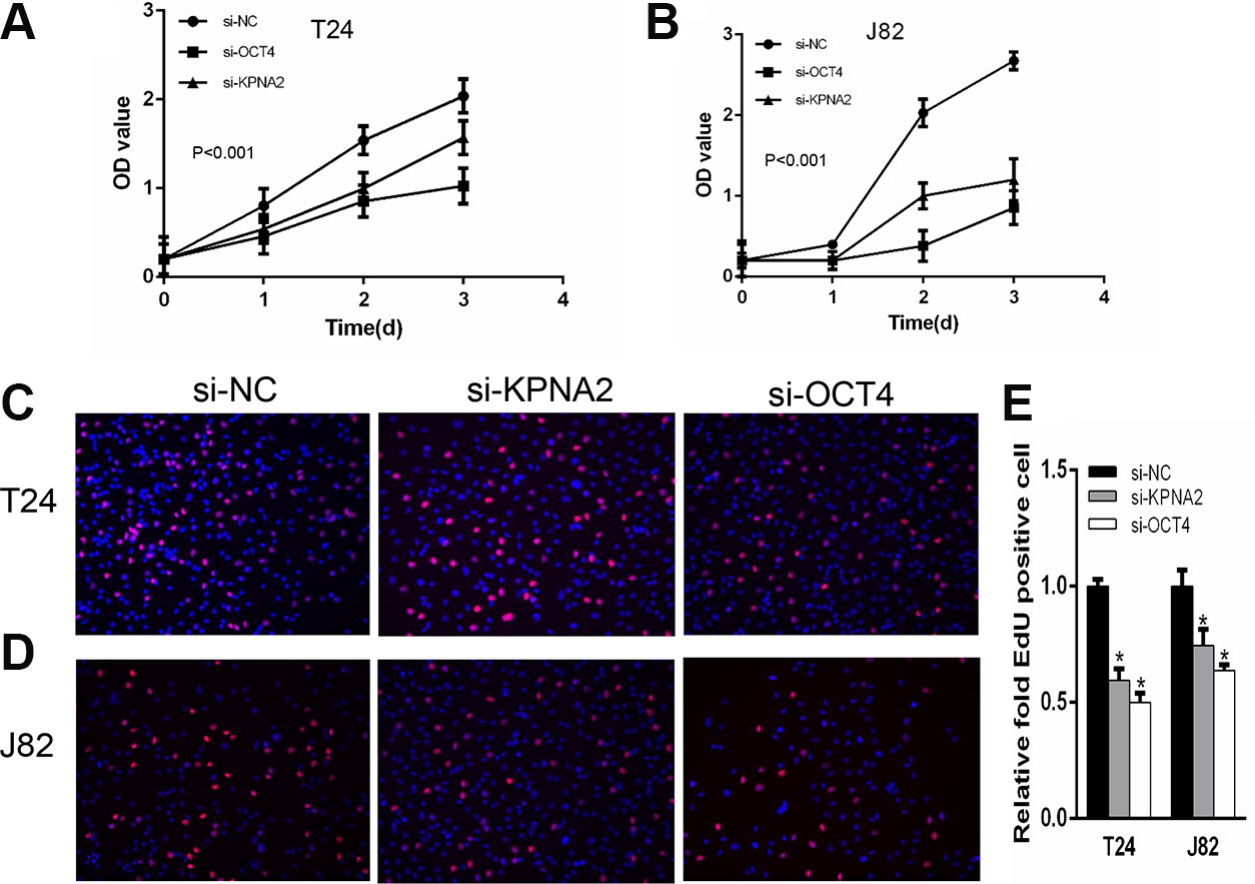
Silencing KPNA2 or OCT4 can inhibit cell proliferation CCK-8 assay was performed to determine cell proliferation rate. (**A**, **B**) KPNA2 and OCT4 siRNA inhibit T24 and J82 cell proliferation. EdU assay was used to confirm the proliferation cells. (**C**, **D**) Cell proliferation was suppressed after transfection with KPNA2 and OCT4 siRNA in T24 and J82 cells. (**E**) EdU positive ratio was quantified to Hoechst 33342 positive cells.

**Figure 4:**
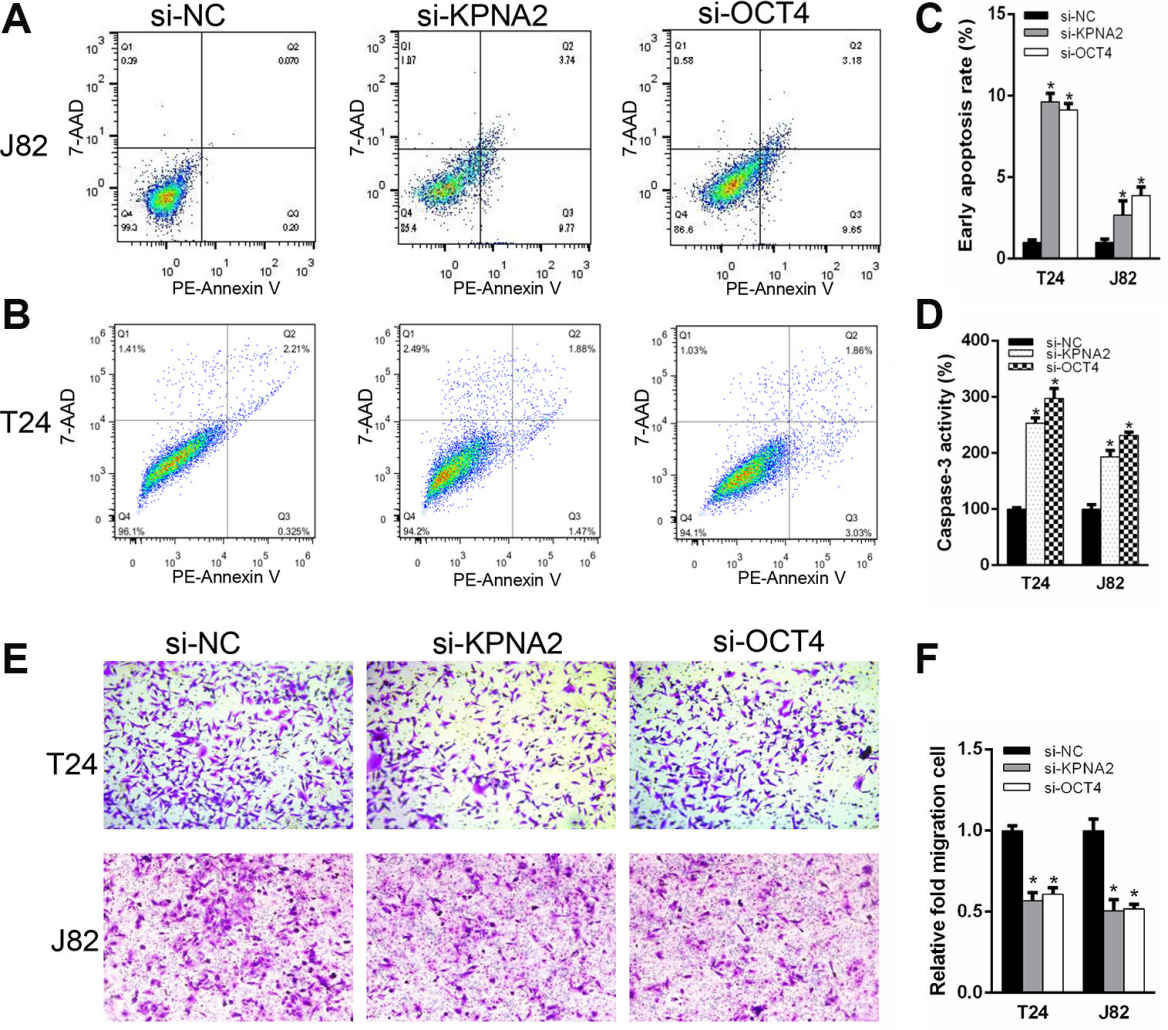
Silencing KPNA2 and OCT4 stimulate cell apoptosis and suppress cell migration ability of the bladder cancer cells Flow cytometry was analyzed to detect early apoptosis rate after transfection with KPNA2 and OCT4 siRNAs compared to negative control. (**A**–**C**) Knockdown the expression of KPNA2 and OCT4, apoptotic cells were increased compared with negative control group in T24 and J82 cell lines. (**D**) Caspase-3 activity was enhanced in the si-KPNA2 and si-OCT4 groups. (**E**, **F**) Transwell assay showed that migration of T24 and J82 cells were inhibited after transfected with KPNA2 and OCT4 siRNAs.

We determined whether KPNA2 and OCT4 influence proliferation in bladder cancer cells. CCK-8 assay was carried out to analyze the proliferation rate of J82 and T24 cell lines after silencing of KPNA2 and OCT4. The results demonstrated that si-KPNA2 and si-OCT4 inhibited cell proliferation remarkably in J82 and T24 cell lines (Figure 3A–3B). Furthermore, we used EdU assay to confirmed cell proliferation rate. As revealed in Figure 3C and 3D, compared to si-NC, EdU positive cells in si-KPNA2 and si-OCT4 groups were significantly decreased in J82 and T24 cells. The relative EdU positive rates of total cells among the siRNA groups were presented in Figure 3E. We investigated whether KPNA2 and OCT4 expression is correlated with the cell apoptosis. Firstly, the apoptosis rate was analyzed using flow cytometry for Annexin-V stained cells (Figure 4A–4C). Moreover, the caspase-3 activity assay was performed to verify the apoptotic changes (Figure 4D). Compared with cells transfected with si-NC, the apoptosis rates and caspase-3 activities were significantly increased in the group of si-KPNA2 or si-OCT4. The results confirmed that both KPNA2 and OCT4 can promote cell proliferation and inhibit cell apoptosis in bladder cancer cells *in vitro*.

### Knockdown of KPNA2 and OCT4 can inhibit cell migration

To test whether KPNA2 and OCT4 can suppress bladder cancer cell migration ability *in vitro*, crystal violet stained cells were calculated after cultured in transwell chambers. A decreased cell motility was observed after transfection of si-KPNA2 and si-OCT4 (Figure 4E). The quantity of migrated cells was demonstrated in Figure 4F. These results showed that KPNA2 and OCT4 siRNAs could decrease bladder cancer cell motility *in vitro*.

### Knockdown of KPNA2 inhibits the nuclear translocation of OCT4

**Figure 5:**
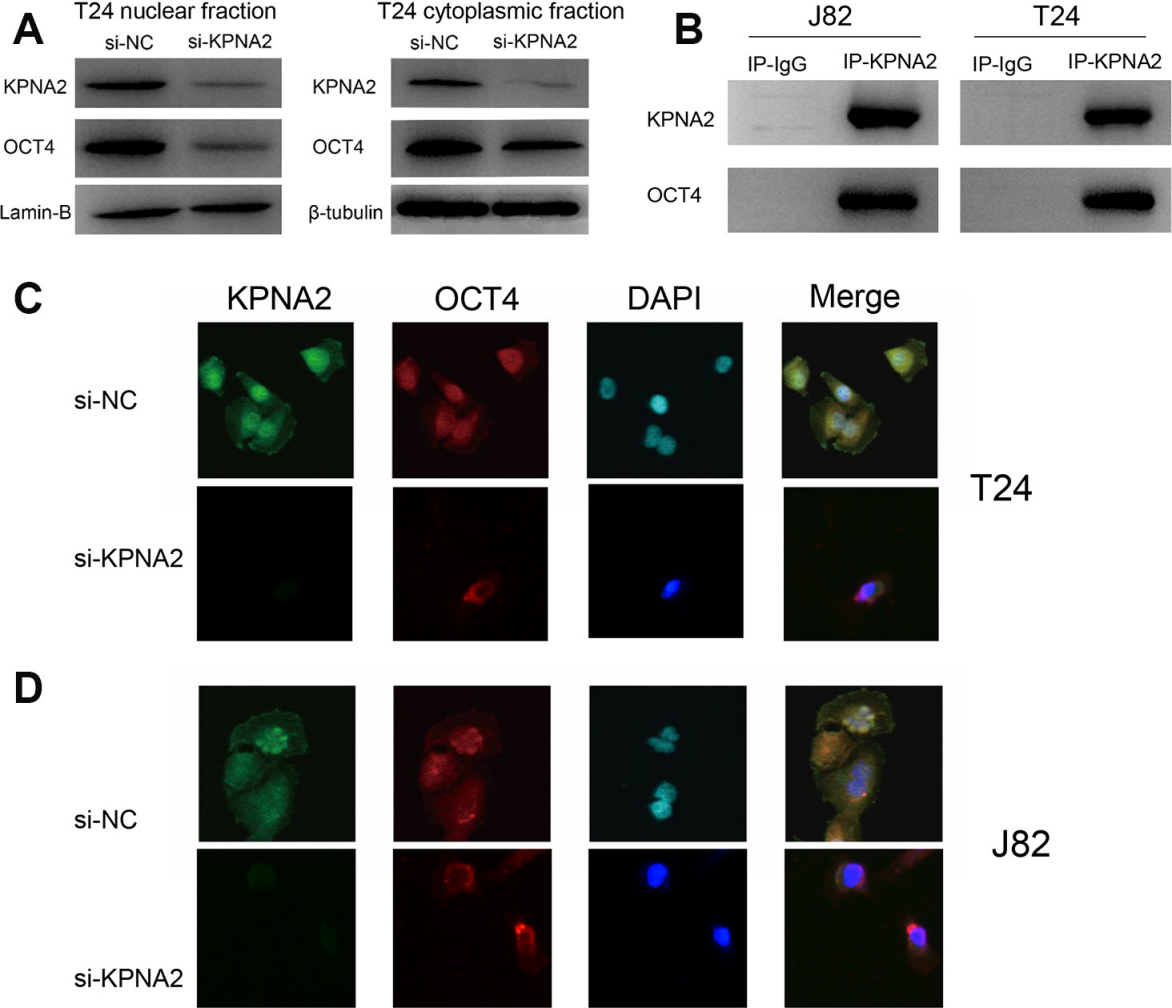
Knockdown of KPNA2 inhibits the nuclear translocation of OCT4 *in vitro* (**A**) After knockdown of KPNA2, OCT4 expression was dramatically decreased in the nuclear fraction of the T24 cell line. (**B**) Co-IP assay was used to examine the association between KPNA2 and OCT4 *in vitro*. (**C**–**D**) The OCT4 fluorescence signal was significantly decreased in cell nucleus as the KPNA2 was silenced in T24 and J82 cell lines.

To investigate whether the process of nuclear translocation of OCT4 is mediated by KPNA2, we detected the expression of OCT4 in subcellular distribution in cytoplasmic and nuclear fractions using western blot. Lamin-B and β-tubulin were used as loading control for nuclear and cytoplasmic fractions respectively. We found that OCT4 was dramatically decreased in nuclear fraction when KPNA2 was silenced. Meanwhile, cytoplasmic OCT4 protein expression level was slightly reduced when compared with the expression in nuclear fraction (Figure 5A). Furthermore, we performed a Co-IP assay to determine whether KPNA2 and OCT4 interact or not in physiological conditions *in vitro* (Figure 5B). We then developed an immunofluorescence assay to confirm the OCT4 subcellular location change when KPNA2 was silenced (Figure 5C and 5D), and found that nuclear OCT4 was decreased in si-KPNA2 group, while cytoplasmic and nuclear OCT4 signals were both shown in si-NC group. These results indicate the association between KPNA2 and OCT4 in bladder cancer cells.

## DISCUSSION

We found that the KPNA2 and OCT4 were expressed in most of the bladder cancer tissues (total no.=195), and that KPNA2 is predominantly a nuclear protein (Figure 1B) and their expression levels were significantly correlated with clinicopathologic characteristics and highly associated with poor prognosis. Moreover, KPNA2 and OCT4 silencing resulted in decreased cell migration ability and proliferation rate and promoted cell apoptosis in bladder cancer cell lines. Co-immunoprecipitation assay showed that OCT4 interacts with KPNA2 in physiological conditions *in vitro*. We found that OCT4 was significantly decreased in nuclear fraction when KPNA2 was silenced. These results indicated that KPNA2 promoted the development of bladder cancer partly through mediating the process of OCT4 nucleocytoplasmic transport.

Abnormal expression level of KPNA2 has been reported in numerous malignancies tumors [14], such as gastric adenocarcinoma, breast cancer and lung cancer [15–19]. KPNA2 is a transduction factor that can mediate signal into cell nucleus and the response of cohesive protein molecules in the cytoplasm exports [20]. Accumulating evidences suggest that KPNA2 contributes to the process of regulate genes, including oncogenes and tumor-suppressor genes, thereby affecting the biological characteristics of cells, such as proliferation, apoptosis and etc. [19, 21–24]. A few of KPNA2-bind cargo proteins have been reported in the tumorigenicity of tumor cells. In FGF1-stimulated cancer cells, KPNA2 was found a role of bounding to FGF1 and FGF2, and the activation of ERK1/2 was increased through ectopic expression of importin α1, finally accelerating the cancer cell proliferation [16]. Among the six members of the importin α family, KPNA2 seems to be the pivotal one for p65 importation. Moreover, in muscle-invasive bladder UC, NF-kappaB p65 nuclear expression parallels histologic grade [25].

OCT4 encodes a transcription factor containing a Pit-Oct-Unc homeodomain, and is a positive regulator of tumor differentiation [26]. Previous study showed that OCT4 could stimulate EMT process in breast cancer [27]. The association between KPNA2 and OCT4 was first reported in the year of 2008 through glutathione S-transferase pull-down assay and Co-IP assay [12]. It is previously being reported that expression of OCT4 is down-regulated with KPNA2 silenced in NSCLC [13]. Our present study is the first report to illustrate the interaction between KPNA2 and OCT4 in bladder cancer. Our findings in the present study are consistent with previously reported studies.

However, our study still has some shortcomings. The follow-up data of bladder patients is insufficient to conclude an explicit relationship between the expression level of KPNA2 and prognosis about bladder cancer patients. Besides, we found that KPNA2 nuclear transport mechanism is an important component of the OCT4 nuclear import process, but the upstream regulatory factor of KPNA2 are needed to be further studied, such as non-coding RNAs. Hsa-miR-26b is reported has a role of directly targets KPNA2. And then, proliferation and migration ability of ovarian carcinoma cells were suppressed by miR-26b/KPNA2/OCT4 axis [28]. We speculate that some non-coding RNAs, such as microRNAs and long non-coding RNAs, may play an important role in the KPNA2 transport mechanism, which needs to be further investigated.

In summary, our data indicates that the aberrant expression of KPNA2 is associated with a poor prognosis and provides the first evidence that KPNA2 contributes to OCT4 nuclear transportation in bladder cancer. Also, we confirmed that up-regulated KPNA2 and OCT4 expression is a common feature of bladder cancer that is correlated with increased aggressive tumor behavior. Additionally, KPNA2 is a pivotal predictor in the development and progression in bladder cancer patients.

## MATERIALS AND METHODS

### Tissue collection and ethics statement

All 195 bladder cancer samples included in this study were obtained from the Peking University Shenzhen Hospital or purchased from US Biomax, Inc. The study was reviewed and approved by the Ethical Committee of Peking University Shenzhen Hospital (Shenzhen, China). The adjacent normal tissue samples were obtained 2.0 cm away from the cancer tissues and confirmed by pathologists. Tissues were formalin fixed and reviewed with hematoxylin & eosin staining to classify histological types and diseased stages. None of the patients received radiotherapy or chemotherapy before this operation.

### Cell culture

The human bladder cancer-derived cell lines (5637, T24, J82, um-uc-3) were obtained from American Type Culture Collection (Manassas, VA, USA). The 5637 cell line was grown in RPMI-1640 Medium (Invitrogen, Carlsbad, CA, USA) supplemented with 10% fetal bovine serum (Gibco). The T24, J82 and um-uc-3 cell lines were cultured in Minimum Essential Medium (Invitrogen, Carlsbad, CA, USA) containing 10% fetal bovine serum (Gibco).

### Western blot analysis

Total cell protein lysates were extracted by RIPA lysis buffer (Thermo Fisher Scientific, Rockford, USA) and boiled at 98°C for 5 min, then separated by 12% SDS-PAGE at 90V for 3 h, sequentially transferred to 0.22 μm PVDF membranes (Millipore). After incubated with specific antibodies overnight at 4°C, these membranes were washed by TBST buffer and incubated with secondary antibodies for 1h at room temperature. Anti-Oct4 and anti-KPNA2 antibodies were purchased from Abcam and Santa Cruz Biotechnology. β-actin and Lamin-B were used as loading control.

### Immunohistochemistry (IHC) and scoring

Tissue microarrays purchased from BioMax were deparaffinized and rehydrated. Antigens on the tissues were retrieved by boiling in citrate buffer (10 mM, PH 6.0). After nonspecific antigens being blocked by normal goat serum, tissue microarrays were incubated with anti-Oct4 antibody (Abcam plc, Cambridge, UK) or anti-KPNA2 antibody (Abcam plc, Cambridge, UK) used at a 1:200 dilution at 4°C overnight. Tissue microarrays were treated with MaxVision UltraSensitive SP (Rabbit)IHC Kit (Maixin Bio, Fujian, China) and stained with DAB stain assay supplied by the kit above. The stain of KPNA2 and OCT4 were scored depend on the staining proportion (on the scale of 1–3: 1: 0%–25%; 2: 25–49%; 3: > 50%) and staining intensity (on a scale of 0–3: negative = 0, weak = 1, moderate = 2, strong = 3). Finally, a comprehensive evaluation scores based on the two results ranging from 0–3. Samples were defined to two groups as follows: the high expression level group (scores: 2–3) and low expression level group (scores: 0–1).

### RNA extraction and real-time PCR

The total RNA of the cultured cells were isolated using Trizol reagent (Invitrogen, Carlsbad, CA, USA). For mRNA analyses, quantitative real-time PCR was performed using a Lightcycler 480 real-time PCR system (Roche Diagnostics GmbH, Mannheim, Germany). Expression levels were normalized with β-actin, as an endogenous control. The mRNA's relative expression levels were determined using ΔΔCt method. The primer sequences were as follows: KPNA2 primers forward: 5′- CTGGGACATCAGAACAAACCAAG-3′, reverse: 5′- ACACTGAGCCATCACCTGCAAT-3′; Oct4 primers forward: 5′-CGCAAGCCCTCATTTCAC-3′, reverse: 5′- CATCACCTCCACCACCTG-3′; β-actin primers forward: 5′-ATAGCACAGCCTGGATAGCAACGTAC-3′, reverse: 5′-CACCTTCTACAATGAGCTGCGTGTG-3′.

### Transfection for bladder cancer cells

Bladder cancer cells T24 and J82 were transfected with 100nM siRNA oligonucleotides using Lipofectamine 3000 followed by the manufacturer's protocol. The siRNA for KPNA2 and Oct4 were ordered from GenePharma, Shanghai, China. The siRNA sequences were listed below: KPNA2-siRNA: (5′-GACUCAGGUUGUGAUUGAUTT-3′ and 5′- AUC AAUCACAACCUGAGUCTT-3′); Oct4-siRNA (5′-CUG GGACACAGUAGAUAGATT-3′ and 5′- UCUAUCUAC UGUGUCCCAGTT-3′).

### CCK-8 assay

The cell proliferation rate change after transfection with specific siRNAs were assessed using the Cell Counting Kit-8 (Dojindo laboratories, Japan). Briefly, a total of approximately 3 × 10^3^ T24 and J82 cells were plated at a 96-well plate. After transfection with KPNA2-siRNA, Oct4-siRNA and negative control siRNA, cells were treated with 10ul/well of Cell Counting Kit-8 solution and incubated for another 1.5 h with total cell culture time point of zero, 24, 48, 72 h. Cell proliferation curves were plotted using the absorbance at a wavelength of 450 nm using Multiskan Go plate reader (Thermo Fisher Scientific Inc, MA, USA). All experiments were performed in triplicate.

### Ethynyl-2-deoxyuridine (EdU) assay

Besides the CCK-8 assay, we performed Ethynyl-2-deoxyuridine (EdU) assay to detect cell proliferation in order to confirm the effect of specific siRNAs. We using a Cell-Light^™^ EdU Apollo^®^ 567 *In Vitro* Imaging Kit (RIBOBIO, Guangzhou, China) following the manufacturer's protocol. A number of 2 × 10^3^ T24 and J82 cells were cultured in 96-well plates for 12h. then transfected with KPNA2-siRNA and Oct4-siRNA. KPNA2 and OCT4 silenced cells were treated with EdU label solution. After fixation with 4% paraformaldehyde, cells were neutralized using glycine. Subsequently, Apollo staining reaction solution was incubated at room temperature and protected from light for 30min to bond the EdU label in cells. After that, cell nucleus was stained with Hoechst 33342 stain solution at room temperature for 30 min. Finally, proliferating rate were calculated using the ratio of the fluorescent positive cells to total cells. Experiments were repeated in triplicate.

### Transwell assay

T24 and J82 cells were harvested after 24 h of transfection with KPNA2-siRNA or Oct4-siRNA. Then 3 × 10^4^ cells were plated on the upper side of the chamber to detect the migratory ability of bladder cancer cells. In the upper side of the transwell chambers, cells were resuspended in serum-free medium while the lower side of the chambers were filled with completed medium (10% FBS) at the same time. After incubation for 24 h at 37°C in the incubator, cells were fixed with 4% paraformaldehyde then stained with 0.1% crystal violet. Stained positive cells in the upper chamber were wiped using cotton swabs. Cells that had invaded and migrated to the membrane were photographed and calculated in four random selected fields per well. Experiments were repeated in triplicate.

### Flow cytometry

The apoptosis rates of T24 and J82 cells after transfection of specific siRNAs were determined using an PE Annexin V Apoptosis Detection Kit I (BD bioscience, USA) according to the manufacturer's protocols. 5 × 10^6^ cells were plated in 6-well plates and transfected with KPNA2-siRNA or Oct4-siRNA respectively. After transfection for 24 h, we harvest cells and resuspend 1 × 10^5^ cells in 100 ul 1× binding buffer after. Each tube was added 5 ul Annexin V-PE stain solution and 5 μl 7-AAD stain solution. Tubes were incubated on ice protected from light for 15 min. Sequentially, early apoptosis frequency were analyzed on a flow cytometry (Beckman flow cytometry FC500). All experiments were repeated for at least three times.

### Caspases-3 activity assay

Caspases are considerable mediators of programmed apoptosis. Among them, caspase-3 is frequently activated and essential for the formation of apoptotic bodies [29]. Thus, caspase-3 activity of T24 and J82 cells was detected after siRNA transfections for 48h using the Human Caspase-3 ELISA Kit (Cusabio, Wuhan, China) according to the manufacturer's instructions to assess cell apoptosis changes.

### Subcellular fractionation location

Separation and preparation of cytoplasmic and nuclear extracts from cultured cells were performed using the NE-PER^™^ Nuclear and Cytoplasmic Extraction Reagents (Invitrogen, Carlsbad, CA, USA) according to the manufacturer's instructions.

### Co-immunoprecipitation (Co-IP)

Co-immunoprecipitation was performed as follows. Briefly, proteins were extracted from T24 and J82 cells with RIPA cell lysis buffer (Invitrogen, Carlsbad, CA, USA). T24 and J82 cell proteins were diluted to 1 μg/μl with PBS and divided into equal amounts. Cell lysates were incubated with protein A/G agarose beads (Life technology) and anti-KPNA2 antibody (Abcam plc, Cambridge, UK) overnight at 4°C. Then centrifuge at 4000 rpm for 30s and wash the pellets carefully pre-chilled RIPA lysis buffer. Finally, binding proteins were boiled to proceed SDS-PAGE. Immunoprecipitates were analyzed with the polyclonal anti-OCT4 antibody (Abcam plc, Cambridge, UK) by Western blot.

### Immunofluorescence assay

To examine the OCT4 subcellular location changes under the circumstance of KPNA2 silencing, we performed indirect immunofluorescence assay. After transfection with KPNA2 siRNA, T24 and J82 cells were fixed using 4% paraformaldehyde and blocked with 5% BSA at room temperature for 60 min. Then cells were incubated with anti-KPNA2 and anti-OCT4 antibody at 4°C overnight. After rinse in PBS buffer for three times, two different fluorochromes conjugated antibodies were incubated with specimen at room temperature for 1 h protected from light. Stained cells were photographed under a fluorescence microscope.

### Statistical analyses

Independent *t* tests or One-way ANOVA test were used to study statistical associations between immunohistochemical data and clinicopathologic parameters. Survival curves for both KPNA2 and OCT4 expression patients were analyzed using the Kaplan-Meier method for statistical significance (version5, GraphPad Software). Relationship between KPNA2 and OCT4 expression level was evaluated using Spearman's correlation coefficient. All analyses were evaluated by SPSS software version 20.0 (IBM Inc, Chicago, USA). The value of P which is less than 0.05 was considered to be stoically significant.
